# Comparison of Fracture Resistance Using Two Different Major Cemented Stems in Osteoporotic Bone Models

**DOI:** 10.3390/jcm14186558

**Published:** 2025-09-18

**Authors:** Kohei Hashimoto, Yukio Nakamura, Nobunori Takahashi, Takkan Morishima

**Affiliations:** 1 Department of Orthopedic Surgery, Aichi Medical University, 1-1 Yazakokarimata, Nagakute 480-1195, Japan; skikohei4145@gmail.com (K.H.); ntakahashi0617@aichi-med-u.ac.jp (N.T.); 2 Department of Orthopedic Surgery, Division of Osteoporosis, Locomotive Syndrome, Joint Disease Center, Aichi Medical University, 1-1 Yazakokarimata, Nagakute 480-1195, Japan

**Keywords:** anterior minimally invasive surgical technique, osteoporotic bone model, periprosthetic femoral fractures, fracture torque and patterns, total hip arthroplasty, biomechanical study

## Abstract

**Background:** Periprosthetic hip fractures (PPFs) are one of the major complications in total hip arthroplasty (THA). Therefore, it is important to identify a mechanism for fracture resistance in THA. This study aimed to clarify the differences in fracture torque and fracture type analysis between two different types of cemented stems. **Methods:** We conducted biomechanical testing of bone analogues using six cemented stems of two different types with osteoporotic bone models: Anterior minimally invasive surgery (AMIS)-K and Charnley-Marcel-Kerboull (CMK) stems. Experienced surgeons implanted each of these types of stems into six bone analogues, and the analogues were compressed and internally rotated until failure. Torque-to-fracture and fracture type were recorded. **Results:** There was no significant difference in fracture torque of AMIS-K stem, compared to the CMK stem (*p* = 0.94). The AMIS-K stem showed one comminuted oblique fracture of Vancouver type B2 and five fractures of type C at the tip of the stem. The CMK stem showed five comminuted oblique fracture of Vancouver type B2 and one of type C at the tip of the stem. Conclusion: The AMIS-K stem demonstrated comparable fracture resistance to the CMK stem in an osteoporotic model.

## 1. Introduction

Anterior minimally invasive surgery (AMIS) is widely used because it limits muscular and capsular disruption and is associated with faster functional recovery [1]. On the femoral side, however, the approach inherently constrains exposure and manoeuvrability, which makes canal preparation more demanding and often necessitates the use of shorter femoral stems to achieve safe broaching and implantation within the restricted working corridor. In cementless total hip arthroplasty (THA), short designs have been shown to mitigate proximal stress shielding and to favourably influence bending behaviour [2]. By contrast, whether “shortness” is advantageous in the cemented setting remains uncertain, because taper-slip constructs rely on a polished tapered stem engaging a uniform cement mantle for controlled subsidence and load transfer—mechanics that differ in principle from ongrowth or ingrowth fixation [2].

The AMIS-K stem was designed on the basis of the stem length and polished tapered configuration of the Charnley–Kerboull femoral stem (Benoist-Girard, Howmedica, Hérouville-Saint-Clair, France), with a shortened overall length and a retained proximal collar to improve compatibility with the AMIS approach. Mechanical testing demonstrated that the 17% shortened model provided favorable results in terms of bending resistance and micromotion, but the 12% shortened model showed the best rotational stability [3]. Based on these comprehensive findings, the 12% shortened stem length was ultimately adopted as the clinical AMIS-K design. Whether these design changes translate to osteoporotic bone, where cortical thickness is reduced and cancellous architecture is weakened, has not yet been established [3]. From a practical standpoint, surgeons using AMIS would benefit from evidence indicating whether a short-collared polished taper can deliver fracture resistance comparable to a longer predecessor without increasing periprosthetic fracture (PPF) risk.

Most prior biomechanical investigations have used young, high-density femora [3], thereby under-representing the fragility profile of osteoporosis [4]. Because PPF remains a major complication after THA with important functional and survival consequences, clarifying its mechanism—and mitigating its risk—requires experiments in models that explicitly simulate osteoporotic bone and that provoke clinically relevant failure patterns [5]. Clinically, cemented THA using the CMK stem has shown a low early PPF rate in patients aged ≥70 years (0.48%) [6], consistent with the taper-slip philosophy, when implantation is meticulous. Furthermore, in normal bone composites the CMK stem has demonstrated greater fracture resistance than a collarless polished tapered (CPT) design and the Versys Advocate stem [7]. Nevertheless, to our knowledge no study has directly compared the short-collared AMIS-K with its longer CMK predecessor in an osteoporotic analogue, leaving a gap in the evidence base relevant to AMIS practice.

With the population ageing, orthopaedic services face a rising burden from osteoporotic (fragility) hip fractures and the concomitant increase in arthroplasty procedures. Recent national-level data show contemporaneous increases in hip fracture numbers and primary hip replacement volume [8]. Multi-country analyses further demonstrate wide variation in age- and sex-standardised incidence (≈95–316 per 100,000) and project that the annual number of hip fractures will nearly double by 2050 (~4.1 million), while 1-year mortality commonly ranges from ~14–28% [9]. In parallel, utilisation of THA for femoral neck fractures has increased in the United States—for example, rising from 8.3% to 13.7% between 2005 and 2014 [10]—and recent registry summaries report that ~27.7% of femoral neck fractures are now treated with THA, with growing adoption of cemented femoral fixation in fracture indications [11,12]. At a health-system level, SCOPE 2021 estimated 4.3 million fragility fractures in the EU27+2 in 2019 with direct costs exceeding €56 billion, yet only ~3% of expenditure was on pharmacologic prevention, underscoring the imperative to shift towards prevention [13]. Because periprosthetic femoral fracture (PPF) treatment is resource-intensive, prevention through optimised implant selection and technique exposure should be prioritised, particularly in osteoporotic bone and approaches with constrained femoral exposure.

We therefore compared, in an osteoporotic composite femur, the torque-to-fracture of the short-collared cemented AMIS-K against the longer CMK under combined axial compression and controlled internal rotation, to provide bench evidence relevant to reducing PPF risk in osteoporotic patients.

## 2. Materials

**Figure 1:**
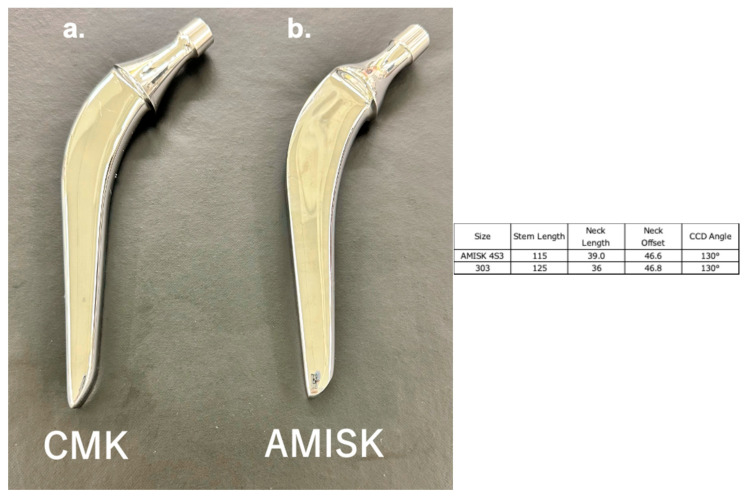
(**a**) Charnley-Marcel-Kerboull stem (Zimmer Biomet). (**b**) AMIS-K stem (Medacta). Stem Length, Neck length and neck offset are expressed in millimetres (mm). Neck offset is measured perpendicular from the stem centreline to the femoral head centre.

We used two types of stems (Figure 1a,b). The CMK 303 stem (Zimmer Biomet, Valence, France) has a polished surface with a collar and a rectangular cross-section. Its double-taper design converts shear stresses into compressive loads, providing stable fixation within the femoral canal. On the other hand, the AMIS-K 4S3 stem (Medacta international S.A., Castel San Pietro, Switzerland) features a double-tapered design and polished surface with a collar and reduced lateral flare. Both stems were manufactured from stainless steel.

One author (TM), an experienced THA surgeon, prepared the constructs by cementing each stem into a left, medium, osteoporotic composite femur analogue (model 3503; Sawbones, Pacific Research Laboratories, Vashon, WA, USA). This model has a thin-walled, low-density cortical shell and 10 pcf cancellous foam (≈0.16 g/cm^3^), which is commonly used to represent osteoporotic bone quality (severe osteoporosis is typically modelled with ≈0.08 g/cm^3^).

The sample size was determined a priori by power analysis based on Morishima et al. (2014): expected difference in fracture torque 40 N·m, SD 20 N·m, α = 0.05, power (1–β) = 0.80, yielding n = 6 per stem type.

For cementation, a distal cement restrictor was placed and a distal centralizer appropriate to each stem was used. Surgical Simplex P PMMA bone cement (medium-viscosity; Stryker Orthopaedics, Kalamazoo, MI, USA) was used for the AMIS-K stem, and Optipack PMMA bone cement (high-viscosity; Zimmer Biomet, Warsaw, IN, USA) for the CMK stem. The polymer powder and monomer liquid were vacuum mixed per IFU; the working/setting time was approximately 10 min at 21 °C. Each stem was inserted at 20° anteversion with neutral varus/valgus, using the central axis of the distal tapered portion as reference. Post-cementation AP and lateral radiographs confirmed alignment within ±1° of the intended position.

### 2.1. Mechanical Loading Test

**Figure 2:**
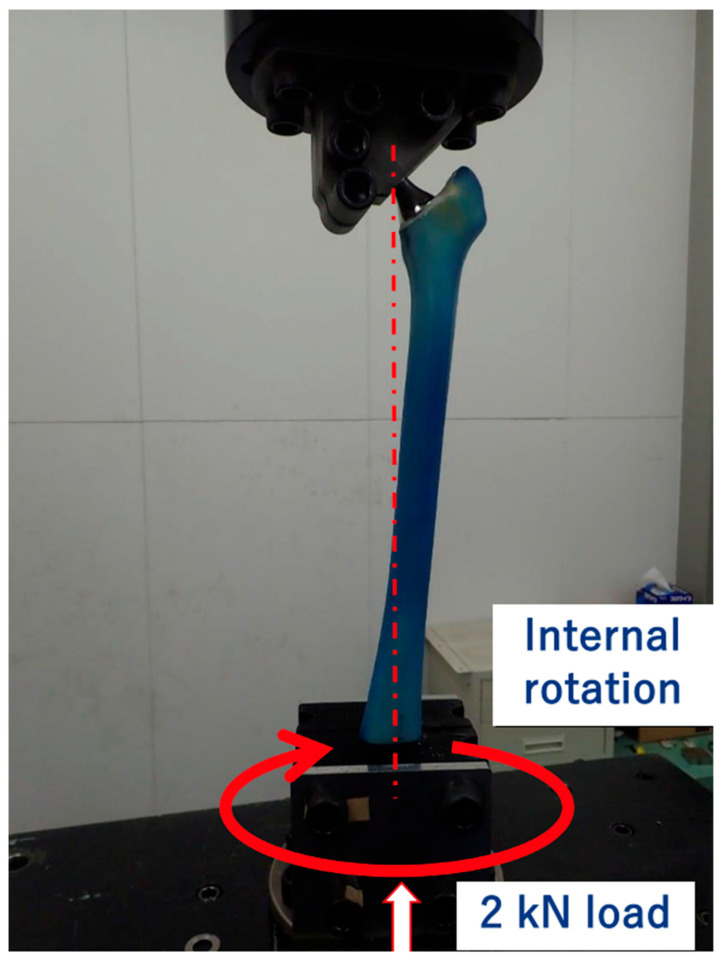
The proximal femur is attached at the centre of rotation of the implant head by means of a clamp. The femoral head is located in the vertical loading axis of the machine, to replicate the natural loading axis of the femur.

We conducted compression–torsion tests using a CMH biaxial material testing system (Saginomiya Seisakusho, Nagoya, Japan). This system enables simultaneous application of axial compression and torsion. The proximal femur was clamped at the centre of rotation of the implant head, and the vertical loading axis of the machine was aligned through the centre of the femoral head and the intercondylar notch. The distal femur was rigidly secured in a custom fixture (Figure 2).

Each construct was tested under combined loading to reproduce fracture patterns in the femur analogue while allowing comparative evaluation between stem designs. A 2 kN axial compressive load and an internal rotation preload of 2 N·m were applied and maintained, and then the implant was internally rotated to 40° over 1 s (i.e., 40°/s) in phase (0° lag) with the axial load, following the methodology of Morishima et al. [14,15]. This angle and rate were chosen to ensure complete fracture in all specimens, which is essential for reliable measurement of maximum torque and consistent pattern analysis under identical, controlled conditions rather than replicating exact in vivo loading scenarios.

Fracture torque was defined as the peak torque recorded at the moment of catastrophic failure. Fracture patterns were classified according to the Vancouver system [16]. Three orthopaedic surgeons independently assessed the patterns, and their ratings were concordant across all specimens. We used six replicates per stem type.

### 2.2. Statistical Analysis

Statistical analyses were performed with JMP Pro 14 (SAS Institute Inc., Cary, NC, USA) for macOS. Because the fracture–torque data did not follow a normal distribution and the per-group sample size was small (n = 6), between-group comparisons used the Mann–Whitney U test (Wilcoxon rank-sum; two-sided). Exact *p*-values were reported, with ties handled by average ranks. The primary endpoint was fracture torque, and statistical significance was set at *p* < 0.05. Box plots display the median (central line), the interquartile range (box), and whiskers extending to 1.5 × IQR; individual points represent observations.

### 2.3. Ethics

No human or animal subjects were involved. All experiments were performed exclusively on synthetic femoral analogues (composite bones); accordingly, approval from an institutional ethics committee (IRB) and informed consent were not applicable.

## 3. Results

### 3.1. Fracture Torque

**Figure 3:**
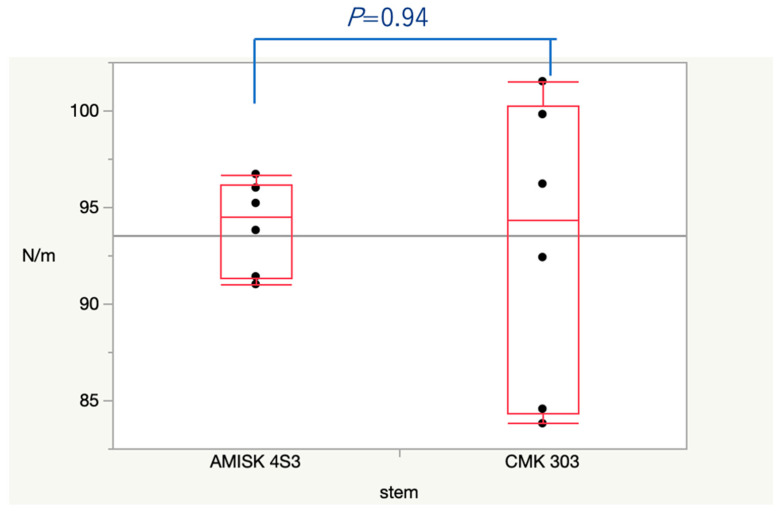
The torque-to-fracture results are presented as box plots. Tests of significance of difference in the means of these results between the two study groups were conducted using the Wilcoxon test. Statistical significance in the difference was denoted when *p* < 0.05. All analyses were conducted using a commercially available software package (JMP^®^ 14 SAS for Macintosh (JMP Statistical Discovery LLC), Cary, NC, USA).

The fracture torques were 93.0 ± 7.6 N·m and 94.0 ± 2.4 N·m when the stems used were CMK 303 and AMISK 4S3, respectively (Figure 3). The difference in fracture torque was not significant (nonparametric test, *p* = 0.94).

### 3.2. Fracture Pattern

**Figure 4:**
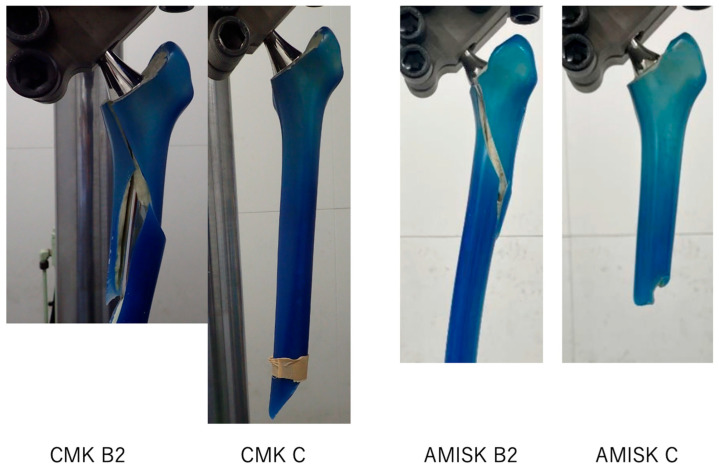
Representative post-failure photographs from the experiment. From left to right: CMK—Vancouver B2 fracture; CMK—Vancouver C fracture; AMIS-K—Vancouver B2 fracture; AMIS-K—Vancouver C fracture.

The fractures were spiral with variable degrees of comminution. In each case, the fracture occurred at the cement–bone interface. There were five comminuted oblique Vancouver type B2 fractures and one type C fracture at the tip of the CMK 303 stem, whereas there was one comminuted Vancouver type B2 fracture and five type C fractures at the tip of the AMISK 4S3 stem (Figure 4).

## 4. Discussion

Periprosthetic femoral fracture (PPF) remains a challenging complication after cemented THA, particularly in osteoporotic bone, and we therefore compared two polished collarless stems that differ primarily in length and proximal volume under identical compression–torsion loading in an osteoporotic femoral analogue. In our model, fracture torque did not differ between AMIS-K and CMK (94.0 ± 2.4 vs. 93.0 ± 7.6 N·m; Mann–Whitney U *p* = 0.94; small effect), while variability was smaller with AMIS-K, which is consistent with the concept that a larger proximal volume may promote more stable metaphyseal load transfer and suggests a potential reduction in sensitivity to minor differences in alignment or cement mantle quality. All constructs failed at the cement–bone interface with spiral fractures; CMK produced predominantly Vancouver B2 patterns (five B2/one C), whereas AMIS-K produced predominantly type C (one B2/five C). This distribution may be compatible with the view that increased proximal stiffness/volume can shift the failure plane distally; however, because AMIS-K is shorter, the increased distance from the isthmus may also have influenced the fracture pattern—further verification is required. Our combined loading (2 kN axial compression with controlled internal rotation) was designed to approximate a single-leg stance during daily activities, enabling consistent failure for comparison. These results refine the prior, partly conflicting literature: Hamadouche et al. reported improved fixation when shortening AMIS-K to the limit of acceptable torque transfer and concluded that shorter stems are beneficial [3], whereas Morishima et al. indicated that shorter stems can present higher fracture risks [14]; our data reconcile these positions by suggesting that shortening per se is not the goal—if a shorter stem is selected, sufficient proximal volume is critical to preserve fracture resistance. With respect to materials, Kaneuji et al. observed greater subsidence in polished taper-slip CoCr stems than in stainless-steel stems, potentially increasing PPF risk via excessive sliding at the stem–cement interface [17], whereas Jain et al. (CPT vs. Exeter) found that neither alloy nor geometry significantly affected resistance to fracture torque in an osteoporotic composite model [18], and Hashimoto et al. suggested that when stem size is appropriately optimised, the influence of alloy composition may be effectively masked [19]. Clinically, PPF is associated with impaired function, subsequent morbidity, and increased mortality [20,21], and osteoporosis is a recognised contributor to PPF risk after THA [22]; taken together with the present findings, AMIS-K may be a reasonable option to mitigate PPF risk in osteoporotic femora, although confirmation in clinical settings is warranted.

On the basis of the present experiments, in older patients with osteoporotic femora, the AMIS-K stem demonstrates fracture resistance comparable to CMK, supporting its clinical use; its shorter length alone should not preclude selection. That said, this single-study design cannot resolve all potential fracture patterns and risks, underscoring the need for broader biomechanical work that encompasses more stem designs. Importantly, both the current bench findings and those from future biomechanical studies require confirmation in clinical studies.

The present study has eight limitations. First, we used a synthetic femoral bone analogue rather than cadaveric femurs. Each has advantages and shortcomings: while synthetic analogues yield highly reproducible results, they do not reproduce the microstructural variability—and therefore the material property variability, notably density—of human femora; conversely, cadaveric femurs are biologically similar to native bone but display substantial between-specimen variability. Second, we evaluated only one length, canal diameter, and shape of the femoral analogue, whereas native femurs vary widely in each of these parameters. Third, we did not include any soft-tissue analogues in the stem–osteoporotic femoral analogue construct. Fourth, for cementation we used PMMA bone cements of different viscosities—a high-viscosity cement for the CMK 303 stem and a medium-viscosity cement for the AMISK 4S3—which may introduce cement-related differences. Fifth, the number of replicates was small (n = 6 per construct type). Sixth, the loading imposed on the construct was quasi-static and comprised only a combination of axial compression and internal-rotation torque; during activities of daily living the stem–native femur combination is subjected to many other, mostly dynamic, loadings. Seventh, only two stem designs were tested, limiting analysis of how specific stem characteristics influence either the torque-to-fracture or the fracture patterns at the cement–bone interface near the proximal tip of the stem. Eighth, we did not quantify the proximal stem volume (or canal occupancy/cement mantle thickness); therefore, inferences regarding the role of proximal volume are qualitative and hypothesis-generating. Future work using CT-based 3D segmentation or CAD-derived mass property analysis is warranted.

## 5. Conclusions

AMIS-K stem showed great fracture resistance, which was equivalent to the CMK stem, suggesting that proximal volume may play a significant role in fracture resistance.

Clinical translation: When femoral exposure is limited—such as with an anterior minimally invasive approach—using a short polished tapered cemented stem with adequate proximal volume need not increase PPF risk. With meticulous rasping, canal preparation, alignment, and cement mantle quality, AMIS-K can be selected with expectations comparable to CMK rather than as a compromise.

## Data Availability

The datasets analysed and presented in this study are available from the corresponding author upon reasonable request.
